# Supplementary material for: A single-cell atlas of West African lungfish respiratory system reveals evolutionary adaptations to terrestrialization
Source: Nat Commun. 2023 Sep 13;14:5630. doi: 10.1038/s41467-023-41309-3 (PMC10497629; doi:10.1038/s41467-023-41309-3)
Supplement: Supplementary file 1 — Supplementary Information [file 41467_2023_41309_MOESM1_ESM.pdf]

Vertebrate respiratory system insights from West African lungfish lung and gill single-cell transcriptomes

Ruihua Zhang[1,2,3,#], Qun Liu[2,3,4,#], Shanshan Pan[2,3,#], Yingying Zhang[2,3], Yating Qin[2,3], Xiao Du[2,3,5], Zengbao Yuan[1,2,3], Yongrui Lu[6], Yue Song[2,3], Mengqi Zhang[2], Nannan Zhang[2,3], Jie Ma[2,3], Zhe Zhang[7], Xiaodong Jia[8], Kun Wang[9], Shunping He[6], Shanshan Liu[5,7], Ming Ni[5,7], Xin Liu[5], Xun Xu[5,10], Huanming Yang[5], Jian Wang[5], Inge Seim[11,12*], Guangyi Fan[2,3,5*]

[1]College of Life Sciences, University of Chinese Academy of Sciences, Beijing 100049, China
[2]BGI Research, Qingdao 266555, China
[3]Qingdao Key Laboratory of Marine Genomics, BGI Research, Qingdao 266555, China
[4]Department of Biology, University of Copenhagen, Copenhagen 2100, Denmark
[5]BGI Research, Shenzhen 518083, China
[6]State Key Laboratory of Freshwater Ecology and Biotechnology, Institute of Hydrobiology, Chinese Academy of Sciences, Wuhan 430072, China.
[7]MGI Tech, Shenzhen, 518083, China
[8]Joint Laboratory for Translational Medicine Research, Liaocheng People's Hospital, Liaocheng 252000, Shandong, P.R. China
[9]Center for Ecological and Environmental Sciences, Northwestern Polytechnical University, Xi'an 710072, China
[10]Guangdong Provincial Key Laboratory of Genome Read and Write, BGI Research, Shenzhen 518083, China
[11]Integrative Biology Laboratory, College of Life Sciences, Nanjing Normal University, Nanjing, China.
[12] School of Biology and Environmental Science, Queensland University of Technology, Brisbane, 4000, Australia
[#]These authors contributed equally.
[*]Correspondence: fanguangyi@genomics.cn (G.F.) and inge@seimlab.org (I.S.).

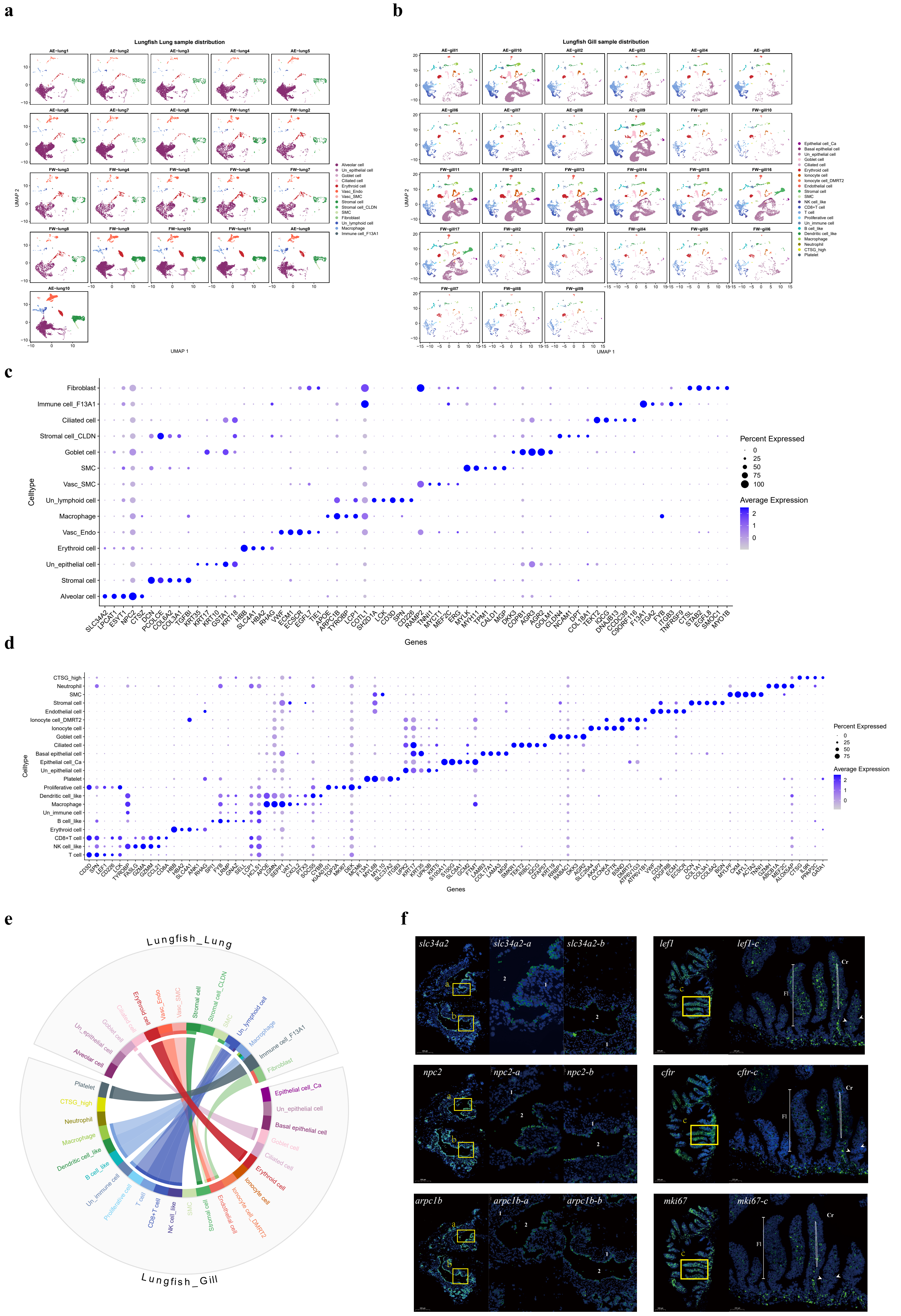
a
b
Lungfish Lung sample distribution
AE-lung1
AE-lung2
AE-lung3
AE-lung4
AE-lung5
AE-lung6
AE-lung7
AE-lung8
FW-lung1
FW-lung2
FW-lung3
FW-lung4
FW-lung5
FW-lung6
FW-lung7
FW-lung8
FW-lung9
FW-lung10
FW-lung11
AE-lung9
AE-lung10
UMAP 1
UMAP 2
Alveolar cell
Un_epithelial cell
Goblet cell
Ciliated cell
Erythroid cell
Vasc_Endo
Vasc_SMC
Stromal cell
Stromal cell_CLDN
SMC
Fibroblast
Un_lymphoid cell
Macrophage
Immune cell_F13A1
Lungfish Gill sample distribution
AE-gill1
AE-gill10
AE-gill2
AE-gill3
AE-gill4
AE-gill5
AE-gill6
AE-gill7
AE-gill8
AE-gill9
FW-gill1
FW-gill10
FW-gill11
FW-gill12
FW-gill13
FW-gill14
FW-gill15
FW-gill16
FW-gill17
FW-gill2
FW-gill3
FW-gill4
FW-gill5
FW-gill6
FW-gill7
FW-gill8
FW-gill9
Epithelial cell_Ca
Basal epithelial cell
Un_epithelial cell
Goblet cell
Ciliated cell
Erythroid cell
Ionocyte cell
Ionocyte cell_DMRT2
Endothelial cell
Stromal cell
SMC
NK cell_like
CD8+T cell
T cell
Proliferative cell
Un_immune cell
B cell_like
Dendritic cell_like
Macrophage
Neutrophil
CTSG_high
Platelet
c
Celltype
Fibroblast
Immune cell_F13A1
Ciliated cell
Stromal cell_CLDN
Goblet cell
SMC
Vasc_SMC
Un_lymphoid cell
Macrophage
Vasc_Endo
Erythroid cell
Un_epithelial cell
Stromal cell
Alveolar cell
Genes
Percent Expressed
0
25
50
75
100
Average Expression
2
1
0
d
Celltype
CTSG_high
Neutrophil
SMC
Stromal cell
Endothelial cell
Ionocyte cell_DMRT2
Ionocyte cell
Goblet cell
Ciliated cell
Basal epithelial cell
Epithelial cell_Ca
Un_epithelial cell
Platelet
Proliferative cell
Dendritic cell_like
Macrophage
Un_immune cell
B cell_like
Erythroid cell
CD8+T cell
NK cell_like
T cell
Genes
Percent Expressed
Average Expression
e
Lungfish_Lung
Lungfish_Gill
f
slc34a2
slc34a2-a
slc34a2-b
lef1
lef1-c
npc2
npc2-a
npc2-b
cftr
cftr-c
arpc1b
arpc1b-a
arpc1b-b
mki67
mki67-c
Fl
Cr

Supplementary Figure 1 | Single cell RNA-seq data composition and features expression, related to Fig1. a, Uniform Manifold Approximation and Projection of lung cell types from single individuals. Annotated as in Fig.1b. **b**, Uniform Manifold Approximation and Projection of gill cell types from single individuals. Annotated as in Fig.1c. **c**, Bubble plot of selected marker genes within each cell type of lung. Color shows the expression levels from grey (low expression level) to blue (high expression level). Circle size reflects the percentage of cells within a cell type which express the specific genes. **d**, Bubble plot of selected marker genes within each cell type of gill. Annotated as in a. **e**, Cell-cell similarities between the lungfish gill and lungfish lung. The top 5% highest Kullback–Leibler divergence (KDL) values are indicated as arches connecting cell types. **f**, Partial enlarged drawing of fluorescence microscopy image of lungfish lung and gills in Fig.1d, e. Each slide is repeated independently 3~5 times. Scalebar, as labeled on the figure.

a

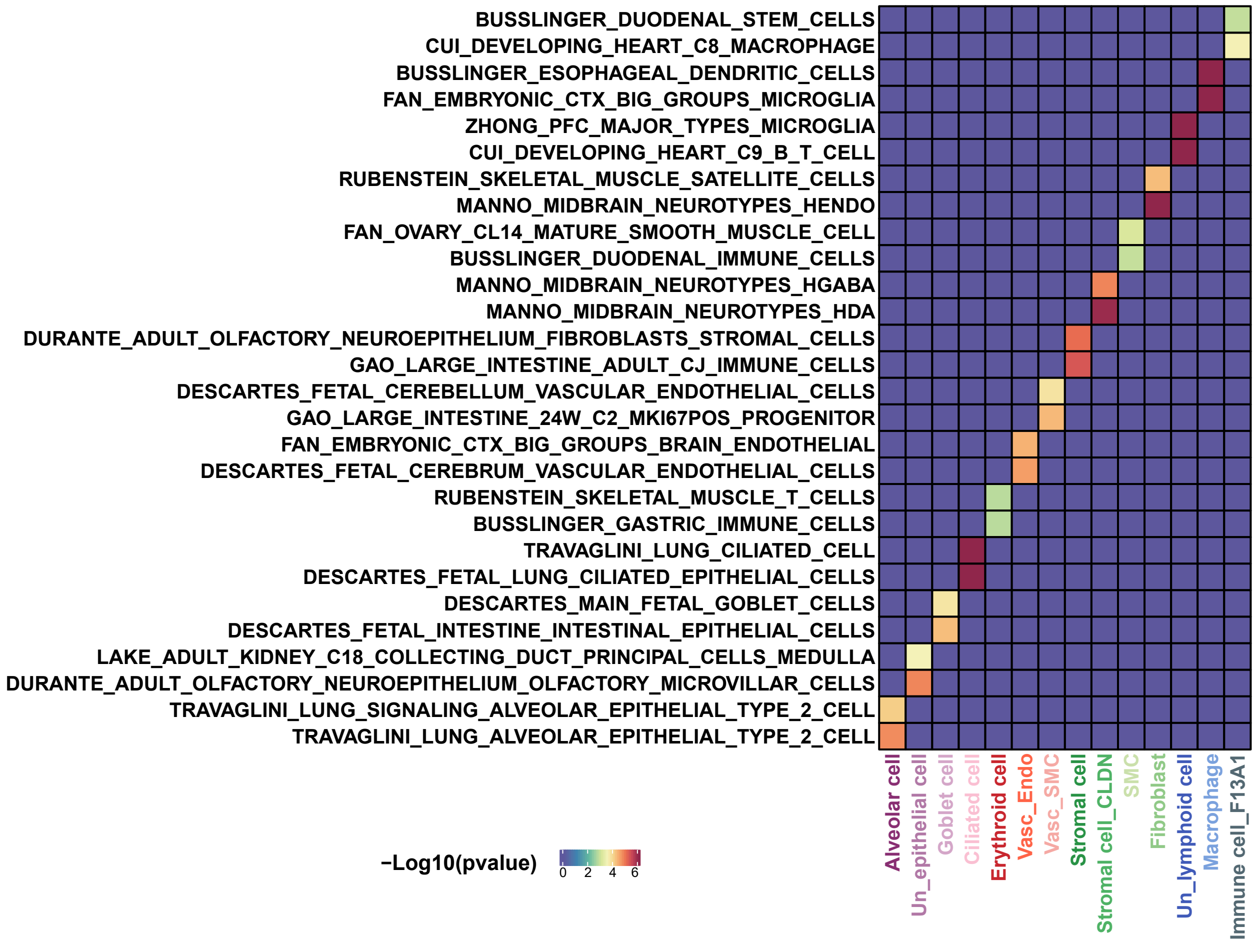

BUSSLINGER_DUODENAL_STEM_CELLS
CUI_DEVELOPING_HEART_C8_MACROPHAGE
BUSSLINGER_ESOPHAGEAL_DENDRITIC_CELLS
FAN_EMBRYONIC_CTX_BIG_GROUPS_MICROGLIA
ZHONG_PFC_MAJOR_TYPES_MICROGLIA
CUI_DEVELOPING_HEART_C9_B_T_CELL
RUBENSTEIN_SKELETAL_MUSCLE_SATELLITE_CELLS
MANNO_MIDBRAIN_NEUROTYPES_HENDO
FAN_OVARY_CL14_MATURE_SMOOTH_MUSCLE_CELL
BUSSLINGER_DUODENAL_IMMUNE_CELLS
MANNO_MIDBRAIN_NEUROTYPES_HGABA
MANNO_MIDBRAIN_NEUROTYPES_HDA
DURANTE_ADULT_OLFACTORY_NEUROEPITHELIUM_FIBROBLASTS_STROMAL_CELLS
GAO_LARGE_INTESTINE_ADULT_CJ_IMMUNE_CELLS
DESCARTES_FETAL_CEREBELLUM_VASCULAR_ENDOTHELIAL_CELLS
GAO_LARGE_INTESTINE_24W_C2_MKI67POS_PROGENITOR
FAN_EMBRYONIC_CTX_BIG_GROUPS_BRAIN_ENDOTHELIAL
DESCARTES_FETAL_CEREBRUM_VASCULAR_ENDOTHELIAL_CELLS
RUBENSTEIN_SKELETAL_MUSCLE_T_CELLS
BUSSLINGER_GASTRIC_IMMUNE_CELLS
TRAVAGLINI_LUNG_CILIATED_CELL
DESCARTES_FETAL_LUNG_CILIATED_EPITHELIAL_CELLS
DESCARTES_MAIN_FETAL_GOBLET_CELLS
DESCARTES_FETAL_INTESTINE_INTESTINAL_EPITHELIAL_CELLS
LAKE_ADULT_KIDNEY_C18_COLLECTING_DUCT_PRINCIPAL_CELLS_MEDULLA
DURANTE_ADULT_OLFACTORY_NEUROEPITHELIUM_OLFACTORY_MICROVILLAR_CELLS
TRAVAGLINI_LUNG_SIGNALING_ALVEOLAR_EPITHELIAL_TYPE_2_CELL
TRAVAGLINI_LUNG_ALVEOLAR_EPITHELIAL_TYPE_2_CELL
-Log10(pvalue)
0 2 4 6
Alveolar cell
Un_epithelial cell
Goblet cell
Ciliated cell
Erythroid cell
Vasc_Endo
Vasc_SMC
Stromal cell
Stromal cell_CLDN
SMC
Fibroblast
Un_lymphoid cell
Macrophage
Immune cell_F13A1

b

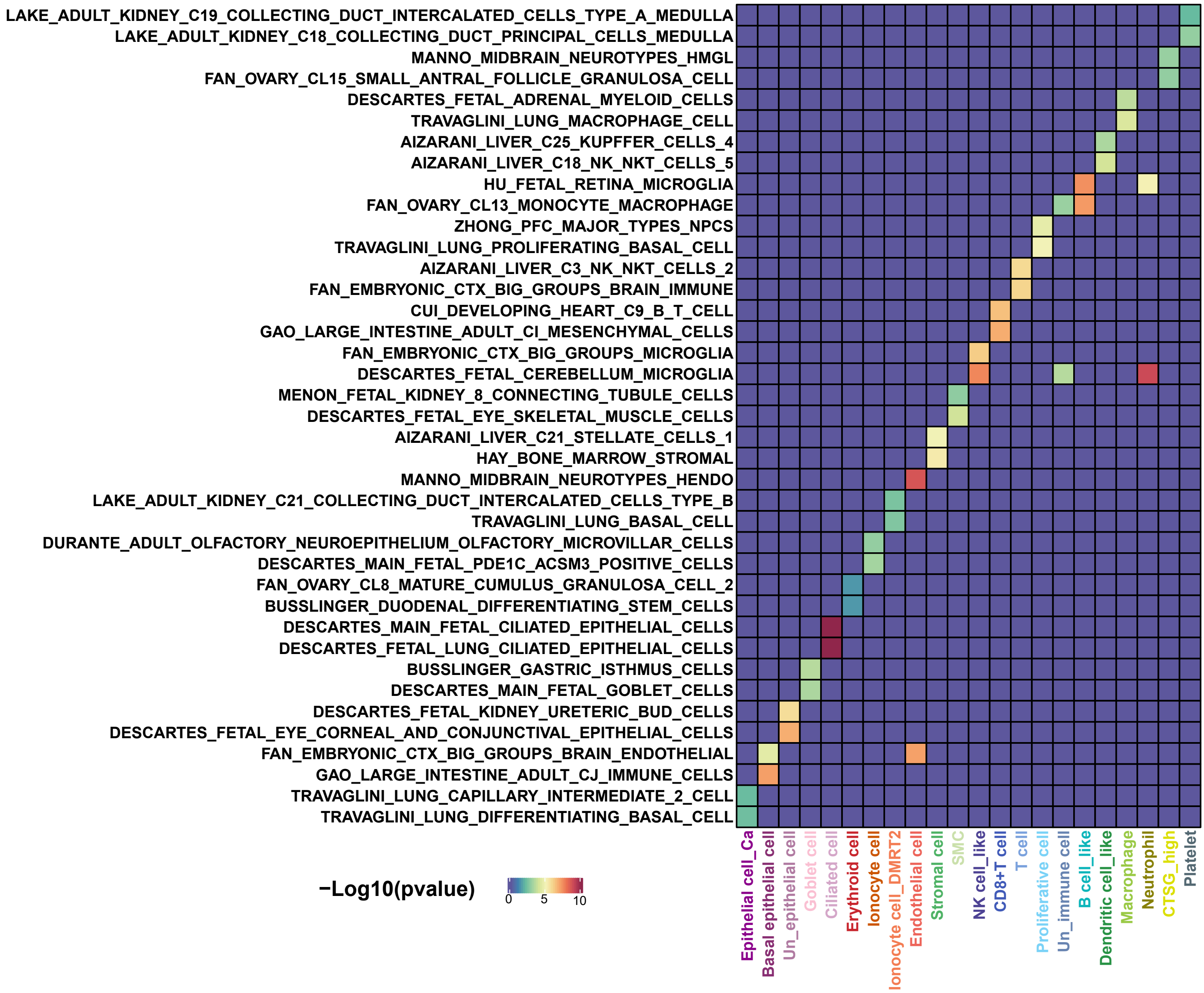

LAKE_ADULT_KIDNEY_C19_COLLECTING_DUCT_INTERCALATED_CELLS_TYPE_A_MEDULLA
LAKE_ADULT_KIDNEY_C18_COLLECTING_DUCT_PRINCIPAL_CELLS_MEDULLA
MANNO_MIDBRAIN_NEUROTYPES_HMGL
FAN_OVARY_CL15_SMALL_ANTRAL_FOLLICLE_GRANULOSA_CELL
DESCARTES_FETAL_ADRENAL_MYELOID_CELLS
TRAVAGLINI_LUNG_MACROPHAGE_CELL
AIZARANI_LIVER_C25_KUPFFER_CELLS_4
AIZARANI_LIVER_C18_NK_NKT_CELLS_5
HU_FETAL_RETINA_MICROGLIA
FAN_OVARY_CL13_MONOCYTE_MACROPHAGE
ZHONG_PFC_MAJOR_TYPES_NPCS
TRAVAGLINI_LUNG_PROLIFERATING_BASAL_CELL
AIZARANI_LIVER_C3_NK_NKT_CELLS_2
FAN_EMBRYONIC_CTX_BIG_GROUPS_BRAIN_IMMUNE
CUI_DEVELOPING_HEART_C9_B_T_CELL
GAO_LARGE_INTESTINE_ADULT_CI_MESENCHYMAL_CELLS
FAN_EMBRYONIC_CTX_BIG_GROUPS_MICROGLIA
DESCARTES_FETAL_CEREBELLUM_MICROGLIA
MENON_FETAL_KIDNEY_8_CONNECTING_TUBULE_CELLS
DESCARTES_FETAL_EYE_SKELETAL_MUSCLE_CELLS
AIZARANI_LIVER_C21_STELLATE_CELLS_1
HAY_BONE_MARROW_STROMAL
MANNO_MIDBRAIN_NEUROTYPES_HENDO
LAKE_ADULT_KIDNEY_C21_COLLECTING_DUCT_INTERCALATED_CELLS_TYPE_B
TRAVAGLINI_LUNG_BASAL_CELL
DURANTE_ADULT_OLFACTORY_NEUROEPITHELIUM_OLFACTORY_MICROVILLAR_CELLS
DESCARTES_MAIN_FETAL_PDE1C_ACSM3_POSITIVE_CELLS
FAN_OVARY_CL8_MATURE_CUMULUS_GRANULOSA_CELL_2
BUSSLINGER_DUODENAL_DIFFERENTIATING_STEM_CELLS
DESCARTES_MAIN_FETAL_CILIATED_EPITHELIAL_CELLS
DESCARTES_FETAL_LUNG_CILIATED_EPITHELIAL_CELLS
BUSSLINGER_GASTRIC_ISTHMUS_CELLS
DESCARTES_MAIN_FETAL_GOBLET_CELLS
DESCARTES_FETAL_KIDNEY_URETERIC_BUD_CELLS
DESCARTES_FETAL_EYE_CORNEAL_AND_CONJUNCTIVAL_EPITHELIAL_CELLS
FAN_EMBRYONIC_CTX_BIG_GROUPS_BRAIN_ENDOTHELIAL
GAO_LARGE_INTESTINE_ADULT_CJ_IMMUNE_CELLS
TRAVAGLINI_LUNG_CAPILLARY_INTERMEDIATE_2_CELL
TRAVAGLINI_LUNG_DIFFERENTIATING_BASAL_CELL
-Log10(pvalue)
0 5 10
Epithelial cell_Ca
Basal epithelial cell
Un_epithelial cell
Goblet cell
Ciliated cell
Erythroid cell
Ionocyte cell
Ionocyte cell_DMRT2
Endothelial cell
Stromal cell
SMC
NK cell_like
CD8+T cell
T cell
Proliferative cell
Un_immune cell
B cell_like
Dendritic cell_like
Macrophage
Neutrophil
CTSG_high
Platelet

Supplementary Figure 2 | Heatmap of top 2 GSEA terms of DEGs in the lung and gill. a, Heatmap of top 2 GSEA terms of DEGs in the lung. GSEA denotes Gene Set Enrichment Analysis; DEG, differentially expressed gene. The color gradient in the legend represents the −log10(p-value). GSEA analysis uses weighted Kolmogorov–Smirnov statistic method. The Benjamini-Hochberg (BH) adjustment method is used by default. Pvalue is lower than 0.05. **b**, Heatmap of top GSEA terms of DEGs in the gill. Annotated as in Fig.1h. Source data are provided as a Source Data file.

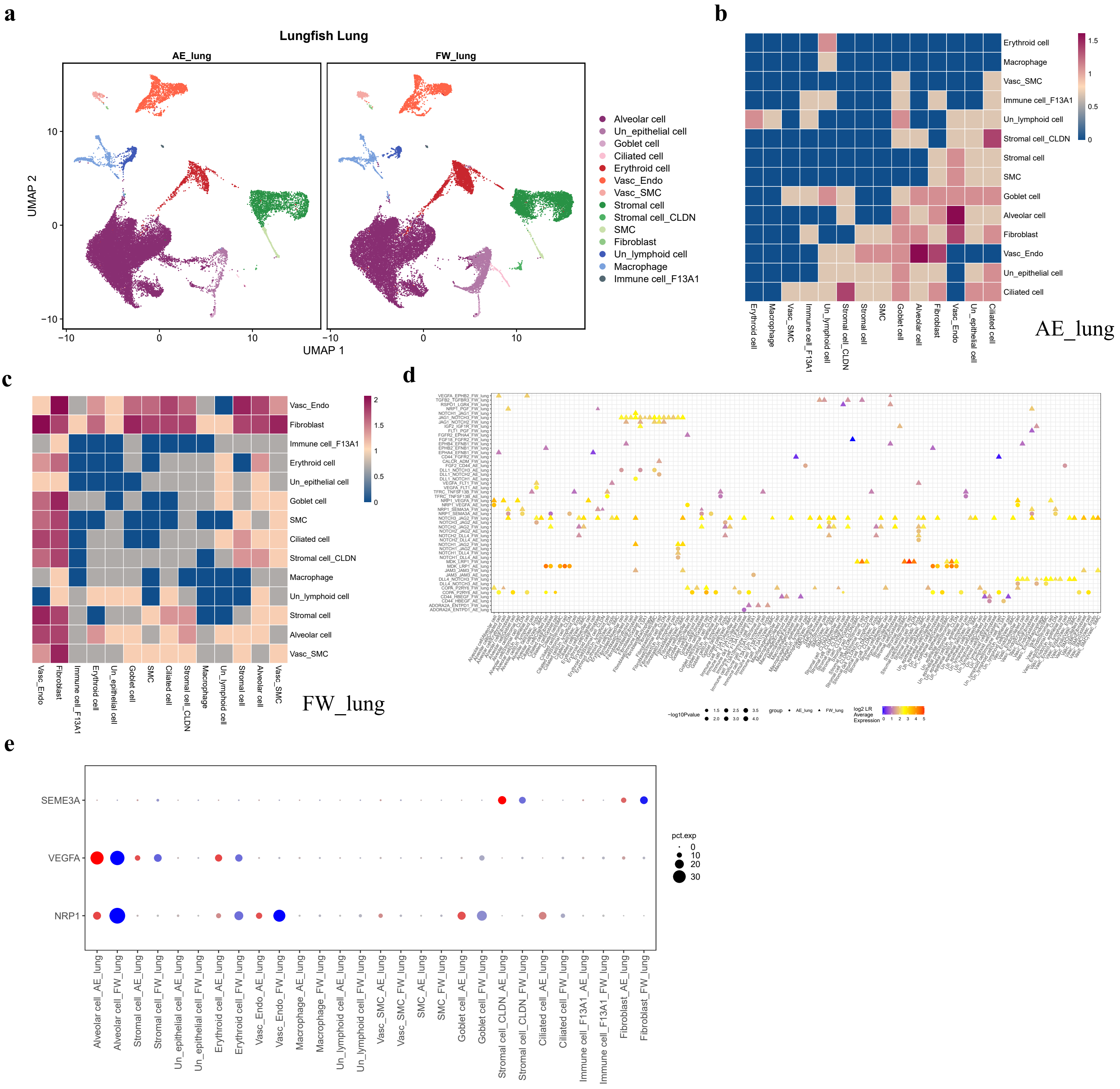

Supplementary Figure 3 | Cellular and molecular features of lungfish lungs during terrestrialization. a, Uniform Manifold Approximation and Projection of lung cell types from single individuals. Cells are color-coded by cluster cell type. Each dot represents a cell, and different colors are associated with specific cell types. b, Heatmap of log-normalized numbers of CellPhoneDB ligand-receptor pairs (LR pairs) between cell types in terrestrialized lungfish lung. c, Heatmap of log-normalized numbers of CellPhoneDB ligand-receptor pairs (LR pairs) between cell types in freshwater lungfish lung. d, Plot showing the expression of all LR pairs in lung cell types from freshwater (FW) and terrestrialized (AE) lungfish. Circle (AE) or triangle (FW) size reflects the percentage of cells within a cell type which express the specific genes. The color of circles/triangles indicates the average expression of the LR pair. Pvalues are calculated by CellphoneDB, which means the likelihood of cell-type specificity of a given receptor–ligand pairs. e, Bubble plot of selected genes in comparisons between freshwater and terrestrialized lung cell types. AE denotes terrestrialization (red bubbles); FW, freshwater (blue bubbles). Circle size reflects the percentage of cells within a cell type which express the specific genes.

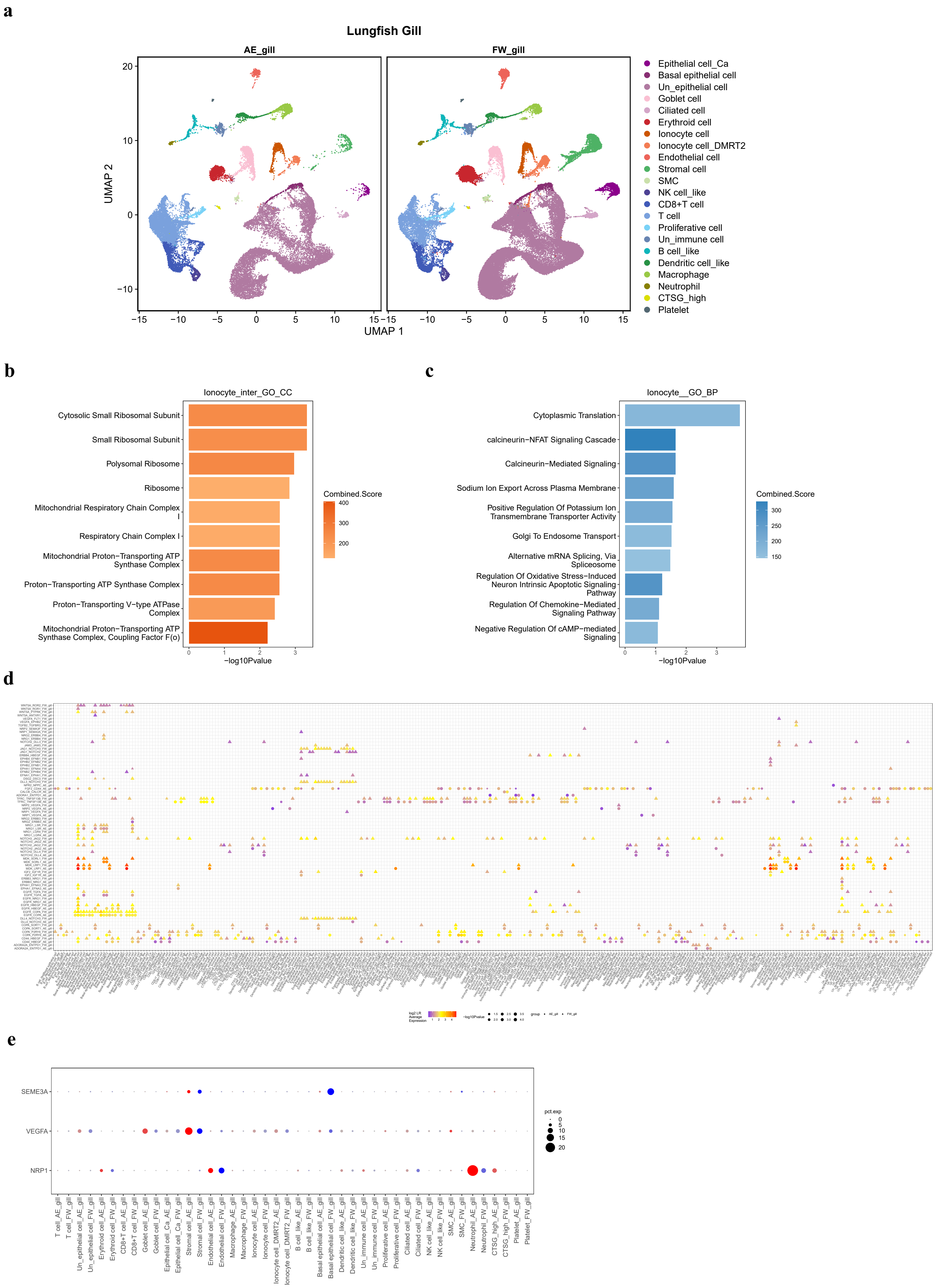
a
Lungfish Gill
AE_gill
FW_gill
UMAP 2
UMAP 1
20
10
0
−10
−15
−10
−5
0
5
10
15
Epithelial cell_Ca
Basal epithelial cell
Un_epithelial cell
Goblet cell
Ciliated cell
Erythroid cell
Ionocyte cell
Ionocyte cell_DMRT2
Endothelial cell
Stromal cell
SMC
NK cell_like
CD8+T cell
T cell
Proliferative cell
Un_immune cell
B cell_like
Dendritic cell_like
Macrophage
Neutrophil
CTSG_high
Platelet
b
Ionocyte_inter_GO_CC
Cytosolic Small Ribosomal Subunit
Small Ribosomal Subunit
Polysomal Ribosome
Ribosome
Mitochondrial Respiratory Chain Complex I
Respiratory Chain Complex I
Mitochondrial Proton−Transporting ATP Synthase Complex
Proton−Transporting ATP Synthase Complex
Proton−Transporting V−type ATPase Complex
Mitochondrial Proton−Transporting ATP Synthase Complex, Coupling Factor F(o)
−log10Pvalue
Combined.Score
400
300
200
c
Ionocyte__GO_BP
Cytoplasmic Translation
calcineurin−NFAT Signaling Cascade
Calcineurin−Mediated Signaling
Sodium Ion Export Across Plasma Membrane
Positive Regulation Of Potassium Ion Transmembrane Transporter Activity
Golgi To Endosome Transport
Alternative mRNA Splicing, Via Spliceosome
Regulation Of Oxidative Stress−Induced Neuron Intrinsic Apoptotic Signaling Pathway
Regulation Of Chemokine−Mediated Signaling Pathway
Negative Regulation Of cAMP−mediated Signaling
−log10Pvalue
Combined.Score
300
250
200
150
d
e
SEME3A
VEGFA
NRP1
pct.exp
0
5
10
15
20
T cell_AE_gill
T cell_FW_gill
Un_epithelial cell_AE_gill
Un_epithelial cell_FW_gill
Erythroid cell_AE_gill
Erythroid cell_FW_gill
CD8+T cell_AE_gill
CD8+T cell_FW_gill
Goblet cell_AE_gill
Goblet cell_FW_gill
Epithelial cell_Ca_AE_gill
Epithelial cell_Ca_FW_gill
Stromal cell_AE_gill
Stromal cell_FW_gill
Endothelial cell_AE_gill
Endothelial cell_FW_gill
Macrophage_AE_gill
Macrophage_FW_gill
Ionocyte cell_AE_gill
Ionocyte cell_FW_gill
Ionocyte cell_DMRT2_AE_gill
Ionocyte cell_DMRT2_FW_gill
B cell_like_AE_gill
B cell_like_FW_gill
Basal epithelial cell_AE_gill
Basal epithelial cell_FW_gill
Dendritic cell_like_AE_gill
Dendritic cell_like_FW_gill
Un_immune cell_AE_gill
Un_immune cell_FW_gill
Proliferative cell_AE_gill
Proliferative cell_FW_gill
Ciliated cell_AE_gill
Ciliated cell_FW_gill
NK cell_like_AE_gill
NK cell_like_FW_gill
SMC_AE_gill
SMC_FW_gill
Neutrophil_AE_gill
Neutrophil_FW_gill
CTSG_high_AE_gill
CTSG_high_FW_gill
Platelet_AE_gill
Platelet_FW_gill

Supplementary Figure 4 | Cellular and molecular features of lungfish gills during terrestrialization. a, Uniform Manifold Approximation and Projection of gill cell types from single individuals. Annotated as in Supplementary Figure 2a. **b**, Enriched GO cellular component of downregulated DEGs of ionocyte cell and ionocyte_DMRT2 cell. The enrichment analysis is generated using Enrichr web server, which take the Fisher exact test, and the Benjamini-Hochberg (BH) was used for correction for multiple hypotheses testing. Pvalues are indicated on the x-axis. **c**, Enriched biological processes of the Ionocyte_Down DEGs. Generated using the Enrichr web server. Annotated as in Supplementary Figure 3b. **d**, Plot showing the expression of all LR pairs in gill cell types of freshwater (FW) and terrestrialized (AE) lungfish. Annotated as in Supplementary Figure 2d. **e**, Bubble plot of selected genes in comparisons between freshwater and terrestrialized gill cell types. Annotated as in Supplementary Figure 2e.

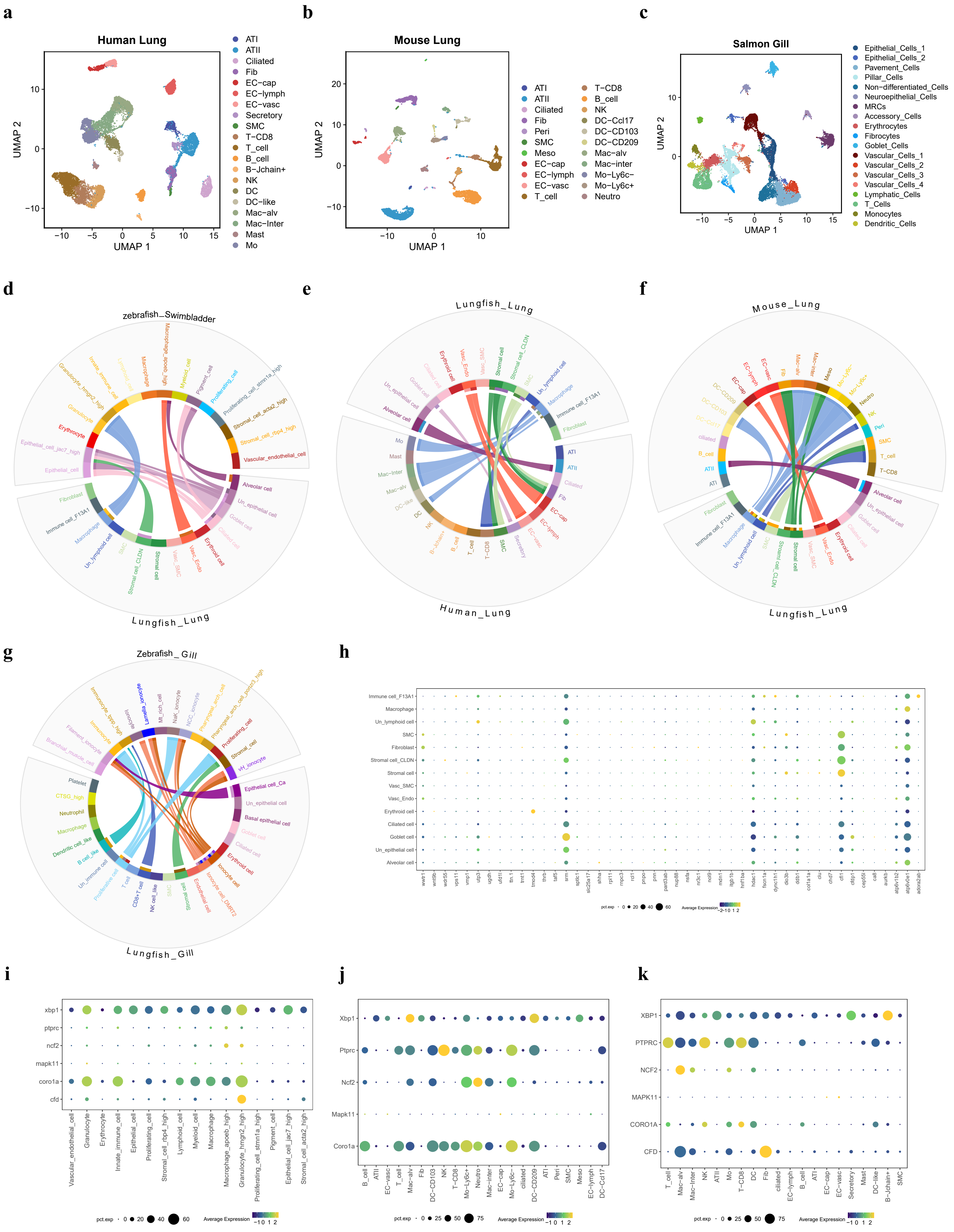

a
Human Lung
UMAP 1
UMAP 2
ATI
ATII
Ciliated
Fib
EC−cap
EC−lymph
EC−vasc
Secretory
SMC
T−CD8
T_cell
B_cell
B−Jchain+
NK
DC
DC−like
Mac−alv
Mac−Inter
Mast
Mo
b
Mouse Lung
UMAP 1
UMAP 2
ATI
ATII
Ciliated
Fib
Peri
SMC
Meso
EC−cap
EC−lymph
EC−vasc
T_cell
T−CD8
B_cell
NK
DC−Ccl17
DC−CD103
DC−CD209
Mac−alv
Mac−inter
Mo−Ly6c−
Mo−Ly6c+
Neutro
c
Salmon Gill
UMAP 1
UMAP 2
Epithelial_Cells_1
Epithelial_Cells_2
Pavement_Cells
Pillar_Cells
Non−differentiated_Cells
Neuroepithelial_Cells
MRCs
Accessory_Cells
Erythrocytes
Fibrocytes
Goblet_Cells
Vascular_Cells_1
Vascular_Cells_2
Vascular_Cells_3
Vascular_Cells_4
Lymphatic_Cells
T_Cells
Monocytes
Dendritic_Cells
d
zebrafish_Swimbladder
Lungfish_Lung
e
Lungfish_Lung
Human_Lung
f
Mouse_Lung
Lungfish_Lung
g
Zebrafish_Gill
Lungfish_Gill
h
i
j
k
pct.exp
Average Expression

Supplementary Figure 5 | Cell type evolution of the lung and gill. a, Uniform Manifold Approximation and Projection of human lung cell types. Cells are color-coded by cluster cell type. Each dot represents a cell, and different colors are associated with specific cell types. **b**, Uniform Manifold Approximation and Projection of mouse lung cell types. Annotated as in a. **c**, Uniform Manifold Approximation and Projection of salmon gill cell types. Annotated as in a. **d**, Cell-cell similarities between the lungfish lung and Atlantic salmon swimbladder. The top 5% highest Kullback–Leibler divergence (KDL) values are indicated as arches connecting cell types. **e**, Cell-cell similarities between the lungfish lung and human lung. Annotated as in d. **f**, Cell-cell similarities between the lungfish lung and mouse lung. Annotated as in d. **g**, Cell-cell similarities between the lungfish gill and zebrafish gill. Annotated as in d. **h**, Bubble plot of 49 orthologous genes form ZFIN database related to phenotype of zebrafish swim bladder. Annotated as in Supplementary Figure 1a. **i-k**, Bubble plot showing the expression of selected DEGs in lungfish-zebrafish-human-mouse comparisons in the zebrafish swim bladder, mouse lung and human lung. Annotated as in Fig.4e.

a

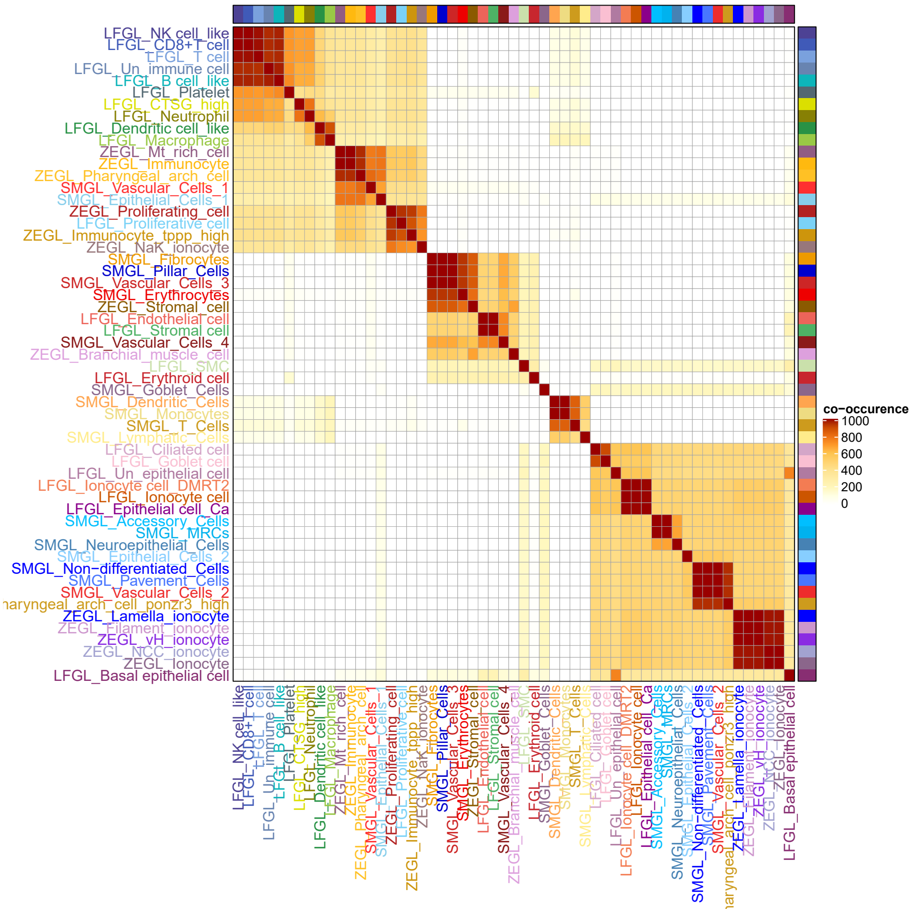

b

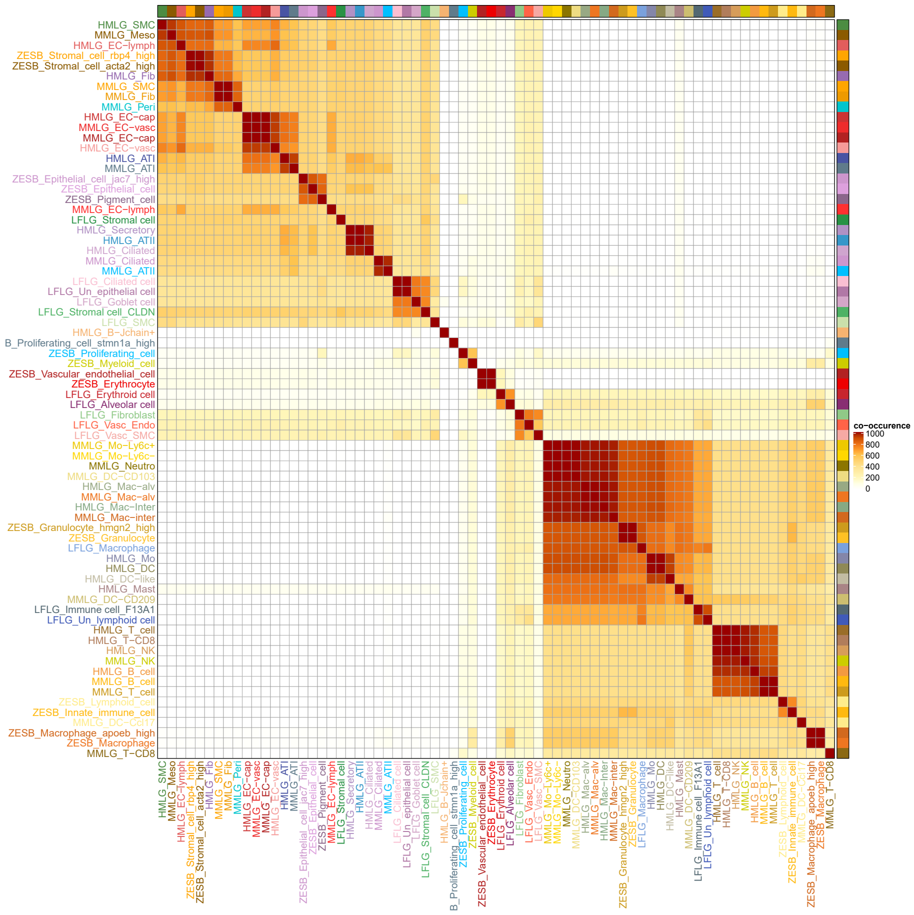

Supplementary Figure 6 | Cell type similarity between species. a, Heatmap showing cell type simi-larity between the gills of the lungfish (LFGL), salmon (SMGL), and zebrafish (ZEGL). Colors in top and right annotations represent different cell types. b, Heatmap showing cell type similarity between lungfish lung (LFLG), mouse lung (MMLG), human lung (HMLG), and zebrafish swim bladder (ZESB). Annotated as in a.
