# Supplementary material for: A single-cell atlas of West African lungfish respiratory system reveals evolutionary adaptations to terrestrialization
Source: Nat Commun. 2023 Sep 13;14:5630. doi: 10.1038/s41467-023-41309-3 (PMC10497629; doi:10.1038/s41467-023-41309-3)
Supplement: Supplementary file 3 — Description of Additional Supplementary Files [file 41467_2023_41309_MOESM3_ESM.pdf]

Description of Additional Supplementary Files Document

Supplementary Data1: QC_statistic for each library.

Supplementary Data2: Celltype DEG of African lungfish gill/lung and cell marker resource.

Supplementary Data3: GSEA result of African lungfish lung and gill cell types.

Supplementary Data4: Numbers of DEGs between aestivated(case) and freshwater(control) African lungfish lung/gill.

Supplementary Data5: DEGs of each cell type between aestivated(case) and freshwater(control) African lungfish lung.

Supplementary Data6: DEGs of each cell type between aestivated(case) and freshwater(control) African lungfish gill.

Supplementary Data7: URLs for scRNA-seq data of other species.

Supplementary Data8: 49 genes from ZFIN.

Supplementary Data9: Gene set of cross-species comparison

Supplementary Data10: DEGs between African lungfish lung (control) and human lung.

Supplementary Data11: DEGs between African lungfish gill (control) and salmon gill.

Supplementary Data12: One to one ortholog genes between African lungfish and other species.

Supplementary Data13: Marker gene probes for FISH experiment.
