# Supplementary material for: A single-cell atlas of West African lungfish respiratory system reveals evolutionary adaptations to terrestrialization
Source: Nat Commun. 2023 Sep 13;14:5630. doi: 10.1038/s41467-023-41309-3 (PMC10497629; doi:10.1038/s41467-023-41309-3)
Supplement: Supplementary file 5 — Reporting Summary [file 41467_2023_41309_MOESM5_ESM.pdf]

nature portfolio

Corresponding author(s): Guangyi Fan

Last updated by author(s): Jul 27, 2023

Reporting Summary

Nature Portfolio wishes to improve the reproducibility of the work that we publish. This form provides structure for consistency and transparency in reporting. For further information on Nature Portfolio policies, see our Editorial Policies and the Editorial Policy Checklist.

Statistics

For all statistical analyses, confirm that the following items are present in the figure legend, table legend, main text, or Methods section.

n/a	Confirmed	
☐	☒	The exact sample size (*n*) for each experimental group/condition, given as a discrete number and unit of measurement
☐	☒	A statement on whether measurements were taken from distinct samples or whether the same sample was measured repeatedly
☐	☒	The statistical test(s) used AND whether they are one- or two-sided<br>*Only common tests should be described solely by name; describe more complex techniques in the Methods section.*
☐	☒	A description of all covariates tested
☐	☒	A description of any assumptions or corrections, such as tests of normality and adjustment for multiple comparisons
☐	☒	A full description of the statistical parameters including central tendency (e.g. means) or other basic estimates (e.g. regression coefficient) AND variation (e.g. standard deviation) or associated estimates of uncertainty (e.g. confidence intervals)
☐	☒	For null hypothesis testing, the test statistic (e.g. *F*, *t*, *r*) with confidence intervals, effect sizes, degrees of freedom and *P* value noted<br>*Give P values as exact values whenever suitable.*
☐	☒	For Bayesian analysis, information on the choice of priors and Markov chain Monte Carlo settings
☐	☒	For hierarchical and complex designs, identification of the appropriate level for tests and full reporting of outcomes
☒	☐	Estimates of effect sizes (e.g. Cohen's *d*, Pearson's *r*), indicating how they were calculated

Our web collection on statistics for biologists contains articles on many of the points above.

Software and code

Policy information about availability of computer code

Data collection	We did not used software for data clooection.
Data analysis	https://github.com/BGI-Qingdao/Lungfish_scRNA_data_analysis

For manuscripts utilizing custom algorithms or software that are central to the research but not yet described in published literature, software must be made available to editors and reviewers. We strongly encourage code deposition in a community repository (e.g. GitHub). See the Nature Portfolio guidelines for submitting code & software for further information.

Data

Policy information about availability of data

All manuscripts must include a data availability statement. This statement should provide the following information, where applicable:

- Accession codes, unique identifiers, or web links for publicly available datasets
- A description of any restrictions on data availability
- For clinical datasets or third party data, please ensure that the statement adheres to our policy

1.The published dataset were downloaded from the listed URLs below:
1)Human and mouse lung scRNA-seq data: https://www.ncbi.nlm.nih.gov/geo/query/acc.cgi?acc=GSE133747
2)Zebrafish gill and swim bladder scRNA-seq data: https://www.ncbi.nlm.nih.gov/geo/query/acc.cgi?acc=GSE130487
3)Atlantic salmon gill scRNA-seq data: https://www.ncbi.nlm.nih.gov/geo/query/acc.cgi

2.The datasets of current study have been uploaded on CNGB: https://db.cngb.org/search/?q=CNP0003631+
3.Genes related to phenotype of zebrafish swim bladder from ZFIN database are available at (http://zfin.org/search?category=Anatomy+%2F+GO&q=swim+bladder)
4.The West African lungfish genome and annotation file are available in the Figshare [https://figshare.com/articles/dataset/The_gff_file_cds_file_and_pep_file_of_the_African_lungfish_genome/13725901]

Research involving human participants, their data, or biological material

Policy information about studies with human participants or human data. See also policy information about sex, gender (identity/presentation), and sexual orientation and race, ethnicity and racism.

Reporting on sex and gender	N/A
Reporting on race, ethnicity, or other socially relevant groupings	N/A
Population characteristics	N/A
Recruitment	N/A
Ethics oversight	N/A

Note that full information on the approval of the study protocol must also be provided in the manuscript.

Field-specific reporting

Please select the one below that is the best fit for your research. If you are not sure, read the appropriate sections before making your selection.

☐ Life sciences ☐ Behavioural & social sciences ☒ Ecological, evolutionary & environmental sciences

For a reference copy of the document with all sections, see nature.com/documents/nr-reporting-summary-flat.pdf

Ecological, evolutionary & environmental sciences study design

All studies must disclose on these points even when the disclosure is negative.

Study description	We used single cell RNA-sequencing on African lungfish (Protopterus annectens) lung and gill.
Research sample	We collected total lung and gill tissue from African lungfish (Protopterus annectens) in freshwater and 33 days terrestrialization state.
Sampling strategy	All the African lungfish (Protopterus annectens) samples (weight 67g~106g, body length ~30cm).
Data collection	The sequencer performed sequencing and transmitted raw sequencing data to the server automatically.
Timing and spatial scale	Start from 2022.3,stop at 2023.5
Data exclusions	There is no data exlcuded from data analysis.
Reproducibility	We used more more than one samples in each group and set technical repetition.
Randomization	random selection
Blinding	*Describe the extent of blinding used during data acquisition and analysis. If blinding was not possible, describe why OR explain why blinding was not relevant to your study.*

Did the study involve field work? ☐ Yes ☒ No

Reporting for specific materials, systems and methods

We require information from authors about some types of materials, experimental systems and methods used in many studies. Here, indicate whether each material, system or method listed is relevant to your study. If you are not sure if a list item applies to your research, read the appropriate section before selecting a response.

Materials & experimental systems

n/a	Involved in the study
x	Antibodies
x	Eukaryotic cell lines
x	Palaeontology and archaeology
	x Animals and other organisms
x	Clinical data
x	Dual use research of concern
x	Plants

Methods

n/a	Involved in the study
x	ChIP-seq
x	Flow cytometry
x	MRI-based neuroimaging

Animals and other research organisms

Policy information about studies involving animals; ARRIVE guidelines recommended for reporting animal research, and Sex and Gender in Research

Laboratory animals	All the African lungfish (Protopterus annectens) samples were in this size (weight 67g~106g, body length ~30cm) and about 2 years old.
Wild animals	Not wild animals.
Reporting on sex	We did not identify the gender for these samples.
Field-collected samples	Not field-collected samples.
Ethics oversight	This study was performed in accordance with the guideline of the national and organizational stipulation. All the process of animal transportation, feeding, the terrestrialization experiment and all aspects of the animal experiments (including animal killing and dissection) were approved by the Institutional Review Board on Ethics Committee of BGI (NO. FT19057-T1).

Note that full information on the approval of the study protocol must also be provided in the manuscript.
